# Rapid structure-based identification of potential SARS-CoV-2 main protease inhibitors

**DOI:** 10.4155/fmc-2020-0264

**Published:** 2021-06-25

**Authors:** M Elizabeth Sobhia, G Siva Kumar, Srikanth Sivangula, Ketan Ghosh, Harmanpreet Singh, Thongtinlal Haokip, Joseph Gibson

**Affiliations:** ^1^Department of Pharmacoinformatics, National Institute of Pharmaceutical Education & Research (NIPER), Sector 67, SAS Nagar, Punjab, 160062, India; ^2^Postgraduate Institute of Medical Education & Research (PGIMER), Sector 12, Chandigarh, 160012, India

**Keywords:** SARS-CoV-2, main protease, water thermodynamics, structure based drug design, drug discovery

## Abstract

The COVID-19 outbreak has thrown the world into an unprecedented crisis. It has posed a challenge to scientists around the globe who are working tirelessly to combat this pandemic. We herein report a set of molecules that may serve as possible inhibitors of the SARS-CoV-2 main protease. To identify these molecules, we followed a combinatorial structure-based strategy, which includes high-throughput virtual screening, molecular docking and WaterMap calculations. The study was carried out using Protein Data Bank structures 5R82 and 6Y2G. DrugBank, Enamine, Natural product and Specs databases, along with a few known antiviral drugs, were used for the screening. WaterMap analysis aided in the recognition of high-potential molecules that can efficiently displace binding-site waters. This study may help the discovery and development of antiviral drugs against SARS-CoV-2.

Since the beginning of the 21st century, the world has witnessed several viral epidemics, including the severe acute respiratory syndrome coronavirus (SARS-CoV) in 2002, the H1N1 influenza virus in 2009 and the Middle East respiratory syndrome coronavirus (MERS-CoV) in 2012 [1]. In December 2019 a novel coronavirus was identified in patients with viral pneumonia in Wuhan, in the Hubei province of China. In Feb 2020 WHO officially named the disease caused by this virus (2019-nCoV) as COVID-19. The virus was further designated as Severe Acute Respiratory Syndrome Coronavirus 2 (SARS-CoV-2) by the International Committee on Taxonomy of Viruses due to its 89.1% nucleotide similarity with the viral genome of SARS-CoV [1,2]. Coronaviruses (CoVs) are enveloped viruses, having a single-stranded, positive-sense RNA genome. They are large spherical viruses with a diameter of 80–120 nm, studded with glycoprotein spikes that give them their characteristic crown-like appearance [3]. CoV infections are common among various other species, including camels, cattle, cats and bats. Tyrell and Bynoe had first reported the virus in 1966 from patients with a common cold [4]. Since then, seven human CoVs have been isolated [5,6] and can be classified into four genera: α, β, γ and δ. The α and β forms are reported to have originated from mammals like bats, while the δ and γ are mostly from pigs and birds [1,7]. SARS-CoV, MERS-CoV and SARS-CoV-2 fall under the β genus of coronaviruses. SARS-CoV-2, like the former two viruses, affects the lower respiratory tract, causing viral pneumonia [8,9]. It is more contagious than SARS-CoV and MERS-CoV and has a basic reproduction number (R^0^) of 2–2.5. With more than 175,000,000 cases and 3,700,000 deaths as of 13 June 2021, this pandemic is posing a severe risk to public health [10].

The RNA genome of CoVs, being one of the largest, is susceptible to repeated recombination processes and thus can produce new strains of different virulence. One-third of the RNA genome codes for the structural proteins: spike (S) protein, membrane (M) protein, envelope (E) protein, nucleocapsid (N) protein, hemagglutinin esterase (HE) glycoprotein and other accessory proteins [11,12]. The remaining two-thirds of the genome is covered by the gene *ORF1a/b*, which produces polyproteins 1a and polyproteins 1ab for viral replication. A further 16 mature nonstructural proteins, including the main protease (M^pro^), arise from further processing of these two polyproteins. Among these nonstructural proteins, Nsp5, or main protease (M^pro^) plays a role in viral replication [13,14]. The substrate-binding pocket of M^pro^ is highly conserved among various CoV genera, which makes it an attractive broad-spectrum antiviral target [15]. Also, the three-dimensional structure of SARS-CoV-2 M^pro^ is highly similar to that of the SARS-CoV M^pro^ as presumed from the 96% sequence identity. Hence it is expected that their catalytic mechanism might be quite similar. The SARS coronavirus M^pro^ is considered to be active in its dimeric form, which helps in the processing of viral polyproteins, thus becoming an attractive target for the discovery of drugs against SARS.

## Catalytic mechanism of the SARS-CoV M^pro^

**Figure 1.**
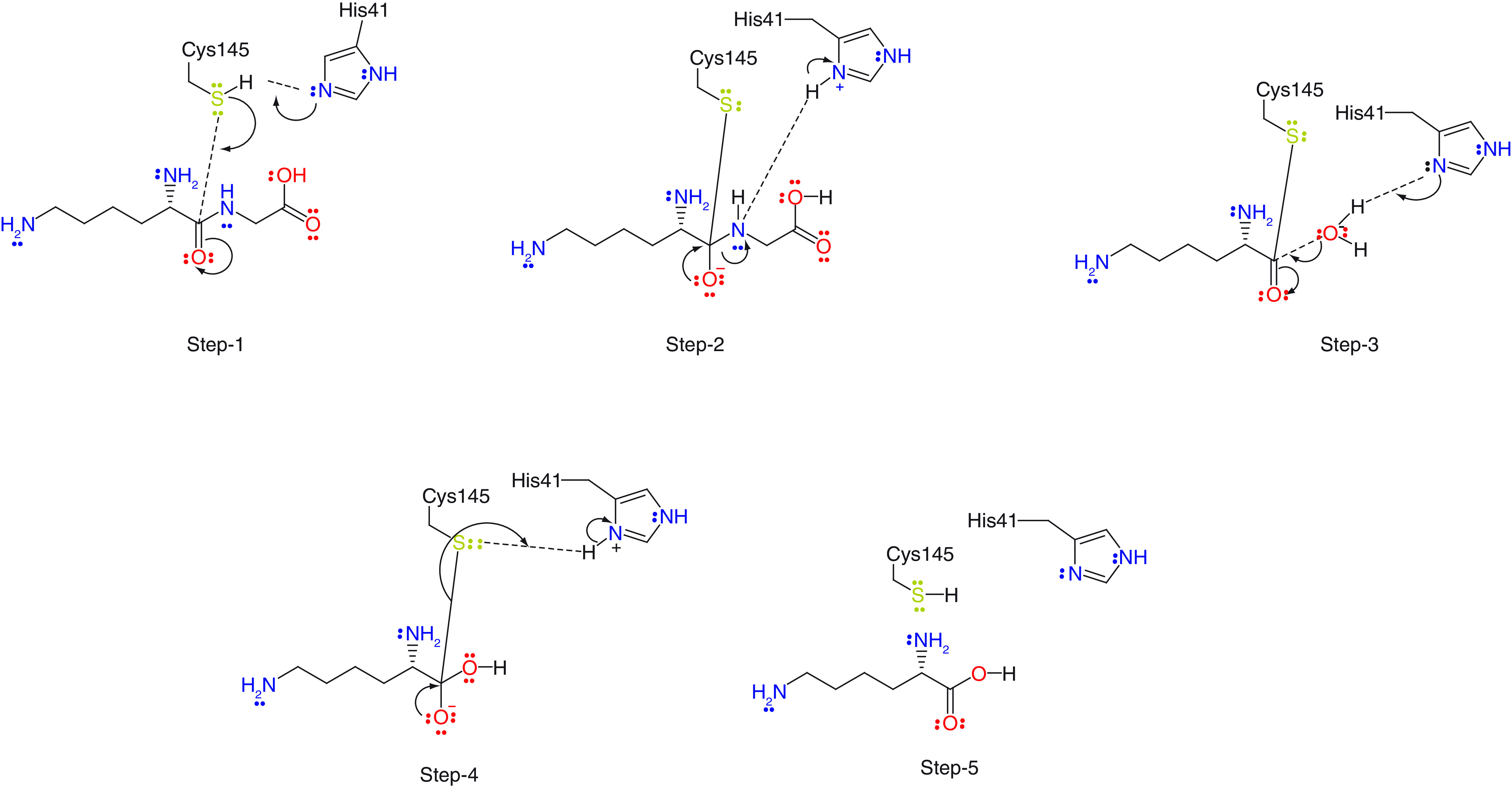
Steps involved in the catalysis of the main protease for viral replication.

The first step in the catalytic mechanism involves the deprotonation and activation of Cys145 by His41, which in turn attacks the carbonyl carbon of the scissile bond through nucleophilic addition. In the second step, an oxyanion generated by His41 triggers the breakage of the peptide bond. The product formed after cleavage acquires a proton at the N-terminal end, from His41. In the third step, His41 is reactivated by accepting a proton from a water molecule. It then attacks the carbon of the thioester bond in a nucleophilic addition. Then in the fourth step, the thioester bond is cleaved by another elimination catalyzed by the oxyanion. Finally, Cys145 accepts a proton from His41 and the enzyme returns to its original state (Figure 1) [16].

The M^pro^ of SARS-CoV-2 is our target of interest for the present study due to various factors. There is an abundance of structural information available on M^pro^ in Protein Data Bank (PDB). It is the main enzyme for viral replication that takes place inside the host cell machinery and it cleaves the overlapping translation products of the virus replicase gene. Therefore inhibiting M^pro^ can lead to the prevention of the proteolytic cleavage processing of the replicase gene, thus blocking the production of infectious viral particles. Moreover, there are no known cellular homologues of M^pro^, which makes it an excellent target for antiviral drug design [17].

The advent of advanced computational technology has paved the way for the application of new methods and approaches in the drug discovery program. In the current COVID-19 pandemic scenario, *in silico* studies have been adopted extensively with the availability of structural information of the virus. With previous success stories of various approved drugs discovered via computational techniques, this gives us a chance to redress the lack of drugs to treat the infection [18]. Researchers are using the latest available PDB crystal structures and are screening approved drug candidates using techniques such as molecular docking, molecular dynamics, virtual screening and pharmacophore mapping. Junmei *et al.* have performed virtual docking screening of drugs including carfilzomib, eravacycline and lopinavir and analyzed their hotspot residues for binding [19]. Khaerunnisa *et al.* screened natural product molecules against the main protease using docking methods [20]. At the same time, Mirza *et al.* have identified potential molecules that would interact with vital SARS-CoV-2 proteins like main protease (M_pro_) and RNA-dependent-RNA-polymerase (RdRp) [21].

## Crystal structure analysis of SARS-CoV-2 M^pro^

On 26 January 2020, the x-ray crystal structure of M^pro^ was deposited in PDB (PDB ID: 6LU7) by Jin *et al.* [22]. Since then, many crystal structures have been resolved, and research groups like Fearon *et al.* and Zhang *et al.* have determined several crystal structures for Mpro of SARS-CoV-2 [23–25]. For the present study, we selected the structures 6Y2G [24] and 5R82 [25]. It was noted that structure 5R82 has a high resolution (1.31 Å) and is bound to a small ligand, whereas 6Y2G (2.2 Å) has a larger ketoamide derivative bound to the active site. The structure of 6Y2G gives a better idea of interactions involved in the binding, especially concerning the catalytic dyad and water molecules near the substrate binding site.

**Figure 2.**
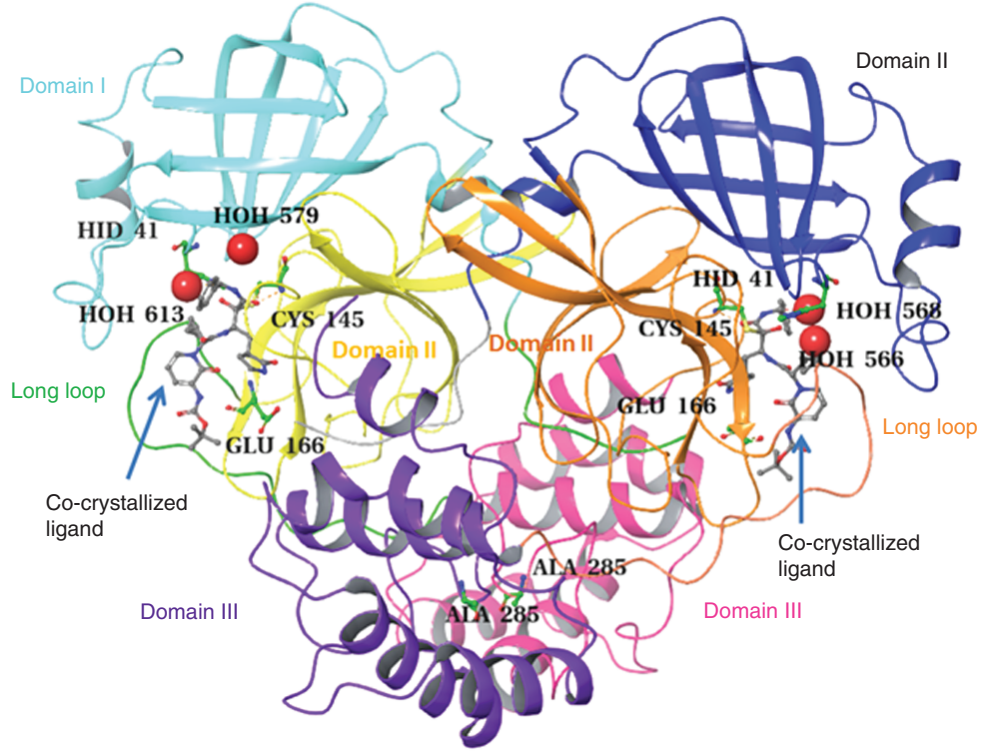
3D structure of the main protease (6Y2G) highlighting protomer A (domain I: cyan; domain II: yellow; domain III: salmon pink), protomer B (domain I: blue; domain II: orange; domain III: plum), Cys145, His41, E166, A285 (green), water molecules (red balls) and long loop (protomer A: green, protomer B: red).

The M^pro^ of SARS-CoV-2 consists of two protomers designated as A and B, each containing three domains: domain I, composed of residues 8–101; domain II, composed of residues 102–184 forming an antiparallel β-barrel structure; and domain III, bearing residues 201–303, which form a cluster of five antiparallel α-helices connected to domain II through a long loop formed of residues 185–200. The substrate binding site of M^pro^, located at the cleft of domains I and II, bears a catalytic dyad of conserved Cys145 and His41 residues (Figure 2) [22].

**Figure 3.**
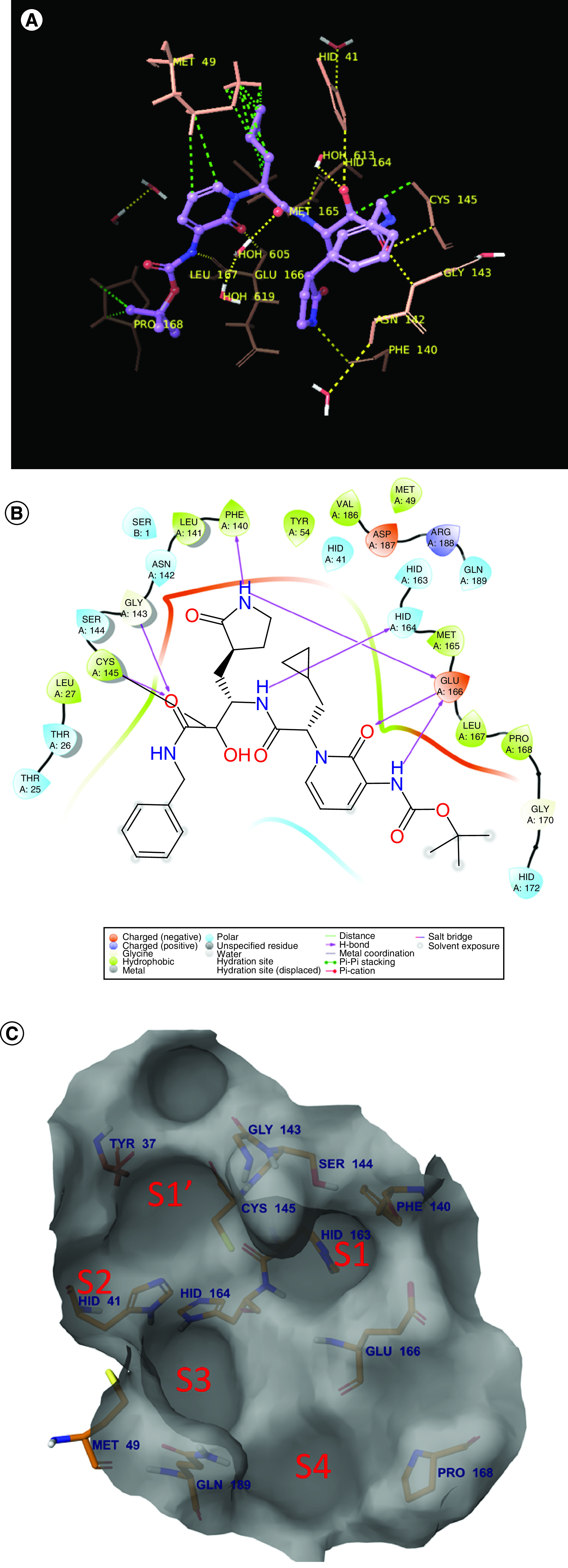
(A) 3D interaction (B) 2D interactions and (C) surface view of the binding site as seen in 6Y2G.

The crystal structure of SARS-CoV-2 M^pro^ (PDB ID: 6Y2G) reveals several important features of the substrate binding pocket. On analysis, it was observed that the binding pocket is neutral and is surrounded by different types of amino acid residues – hydrophobic (Phe140, Leu141, Cys145, Pro168, Leu167, Met165, Cys44), polar (Asn142, Ser144, His41, Thr26, Thr25, Gln189, Gln192, His163, His164), negatively charged (Glu166, Asp187) and positively charged (Arg188) – with two ordered water molecules (HOH613, HOH605) (Figure 3A & B). Also, the entire binding site is divided into different subpockets, namely S1, S2, S3, S4, S1′ (Figure 3C); the molecules that would be docked should engage into this pocket to make the vital interactions with the catalytic dyad and other important residues.

The crystal structure reveals that the cocrystallized ligand interacts with the protein via hydrogen bonds at His41, Cys145 (catalytic dyad residues), Phe140, Gly143, Ser144, His163, His164 and Glu166, which are indicated with yellow dots in Figure 3A). In addition, it also displayed a covalent interaction with Cys145. The ligand also makes hydrophobic interactions with residues Met49, Met165 and Cys145, shown in green dotted lines in Figure 3A.

The focus of the present study is to use computational approaches to identify potential drug-like molecules that can inhibit the M^pro^ of SARS-CoV-2. We followed a fast mode approach by using a combinatorial and structure-based strategy to achieve this goal; at the same time, we followed several stringent criteria for the selection of molecules.

## Materials & methods

### 3D-Databases preparation

Compounds identified from four databases, namely Enamine (www.enaminestore.com), Specs (www.specs.net), Natural product [26], DrugBank [27] and several antiviral drugs, including hydroxychloroquine, were refined by eliminating duplicates and adding missing hydrogen atoms. Then all the databases were refined using the LigPrep module of the Schrödinger suite [28]. The chiralities of ligands were preserved and a minimum of five low-energy stereoisomers per ligand were generated, using default conditions at pH 7.0 ± 2.0. The Epik utility was used for accurate enumeration of ligand protonation states in biological conditions.

### Protein preparation

The proteins were prepared using the Protein Preparation Wizard utility in Maestro [29]. The structure preparation involved adding hydrogen atoms, optimizing the hydrogen bonds, removing atomic clashes, adding formal charges to the hetero groups and then optimizing it at neutral pH. An Optimized Potential for Liquid Simulations-3e (OPLS-3e) force field was used to minimize the prepared structures [30]. The important crystal water molecules near the catalytic site and substrate binding site were retained for docking studies.

### Receptor interaction grid generation

The centroid of bound ligand in the crystal structure 6Y2G was used for the receptor grid generation. The grid box was extended in all directions to cover the entire binding site. No constraints were used while preparing the grid.

### Molecular docking

Docking studies were performed using the Glide module of the Schrödinger suite [31]. A three-tiered docking strategy was employed on the prepared ligands, consisting of high-throughput virtual screening (HTVS), standard precision (SP) and extra precision (XP). The top molecules, screened through crude rapid HTVS docking, were passed on to the second stage of SP docking. Molecules screened through SP docking were then docked using the more accurate and computationally intensive XP mode. The HTVS and SP docking protocols use the same scoring function; however, HTVS reduces the number of intermediate conformers throughout the docking and reduces the thoroughness of the final torsional refinement and sampling. On the other hand, the XP protocol does more extensive sampling than SP and employs a scoring function that is more effective than SP Glide Score, with higher requirements for ligand–receptor shape complementarity. Flexible ligand sampling was used for all three docking modes. Epik state penalty was applied if the ligands did not fit the conformation of the receptor used. No functional group constraints or torsional constraints were applied during the docking process. Post-docking minimization was performed for the ligands, and all other parameters were set to default when following the three-tiered docking strategy.

### Prime molecular mechanics–generalized Born surface area

The molecular mechanics–generalized Born surface area (MM-GBSA) method was used as a post-docking analysis tool. The variable dielectric generalized Born solvation method (VSGB) and other default parameters were used for the MM-GBSA calculation [32]. Based on the docked complex, this calculates the binding free energy (ΔG_bind_) of each ligand according to the following equation:

$$\Delta {\text{G}}_{\text{bind}}=\Delta {\text{E}}_{\text{intra}}+\Delta {\text{G}}_{\text{solv}}-\text{T}\Delta {\text{S}}_{\text{conf}}+{\text{E}}_{\text{vdW}}+{\text{E}}_{\text{elect}}+{\text{E}}_{\text{ptn}}$$

where ΔE_intra_ and ΔG_solv_ terms represent the intramolecular strain and the desolvation penalties for each ligand upon binding, respectively; −TΔS_conf_ represents the product of ligand conformational entropy penalty and temperature*;* E_vdW_ and E_elect_ depict intermolecular van der Waal’s and electrostatic interaction energies between protein and ligand; and E_ptn_ represents protein energy obtained after minimization. The MM-GBSA scores combined with WaterMap scores were used while selecting the final set of molecules.

### WaterMap calculations

WaterMap is based on molecular dynamics simulations and is used for describing the thermodynamic properties of water molecules interacting with the protein environment. It classifies water hydration sites into two categories, namely stable and unstable waters, based on the thermodynamic properties like entropy (−TΔS), enthalpy (ΔH), free energy (ΔG). Stable water denotes hydration sites with negative free energy, while unstable water denotes hydration sites with positive free energy. In general, hydration sites with high positive values will provide directions to improve the selectivity and potency of a ligand. The WaterMap is composed of a pre-existing cavity in the solution that is created to hold the ligand. The water molecules at the binding site are moved to the bulk solution to make space for ligand binding, leaving a cavity of similar size in the protein. The free energy of waters displaced from the binding site to the bulk solution is estimated using the displaced solvent function of WaterMap. The prepared proteins for both 5RH2 and 6Y2G were used for WaterMap calculation using the default parameters. The thermodynamic and structural properties of theoretical water molecules (hydration sites) in the binding site of 6Y2G were obtained from MD simulation of 2 ns using WaterMap [33].

### Absorption, distribution, metabolism and excretion prediction

A set of absorption, distribution, metabolism and excretion (ADME)-related properties (46 molecular descriptors) was calculated for the selected molecules using the QikProp module [34]. This calculation generates physically relevant descriptors, and the data are used in performing ADME predictions. An overall ADME compliance score drug-likeness parameter (indicated by a number of stars) was used to assess the pharmacokinetic profiles of the compounds. Other parameters like molecular weight (MW), predicted octanol / water partition coefficient (QPlogPo/w), predicted apparent Caco-2 cell permeability (QPPCaco), predicted brain/blood partition coefficient (QPlogBB ), predicted human oral absorption (PHOA) and Lipinski's rule of five (ROF) were also calculated.

## Results & discussion

A fast-mode structure-based drug design approach was followed to screen molecules from four different small-molecule libraries. After structural analysis of the several main protease complexes, two crystal structure complexes (PDB IDs: 5R82 and 6Y2G) were chosen for further computational studies. For the molecular docking studies, the 6Y2G structure bound to a larger inhibitor was selected. The reference ligand of 6Y2G was docked into the binding site using Glide XP docking that reproduced the crystallographic conformation of the ligand. A total of 778,498 molecules from databases (Enamine, Natural product, Specs and DrugBank) were analyzed by following the three-tiered docking strategy described above. All the molecules were docked using the HTVS protocol, which yielded 1000 top-scoring molecules, and these molecules were docked again using the SP protocol. The 500 top-scoring molecules obtained from SP docking were then docked again using the more accurate and computationally intensive XP docking protocol. The top-scoring molecules from each database obtained after XP docking were then selected for MM-GBSA and WaterMap calculations. Finally, we had 362 molecules in hand to choose from, ranked according to the combined WaterMap/MM-GBSA scoring protocol (WM/MM) binding free energies. Both the data obtained from calculations and our prior knowledge were utilized to analyze the results, and we followed specific criteria for the selection of hits. The factors considered in the final selection of hits were WM/MM scores, binding pattern, key protein–ligand interactions and binding pose of the molecule compared with the reference ligand. The analysis indicated that the major residues with which the ligand should preferably interact to inhibit the enzyme were His41, Cys145, Phe140, Gly143, Ser144, His163, His164 and Glu166.

**Figure 4.**
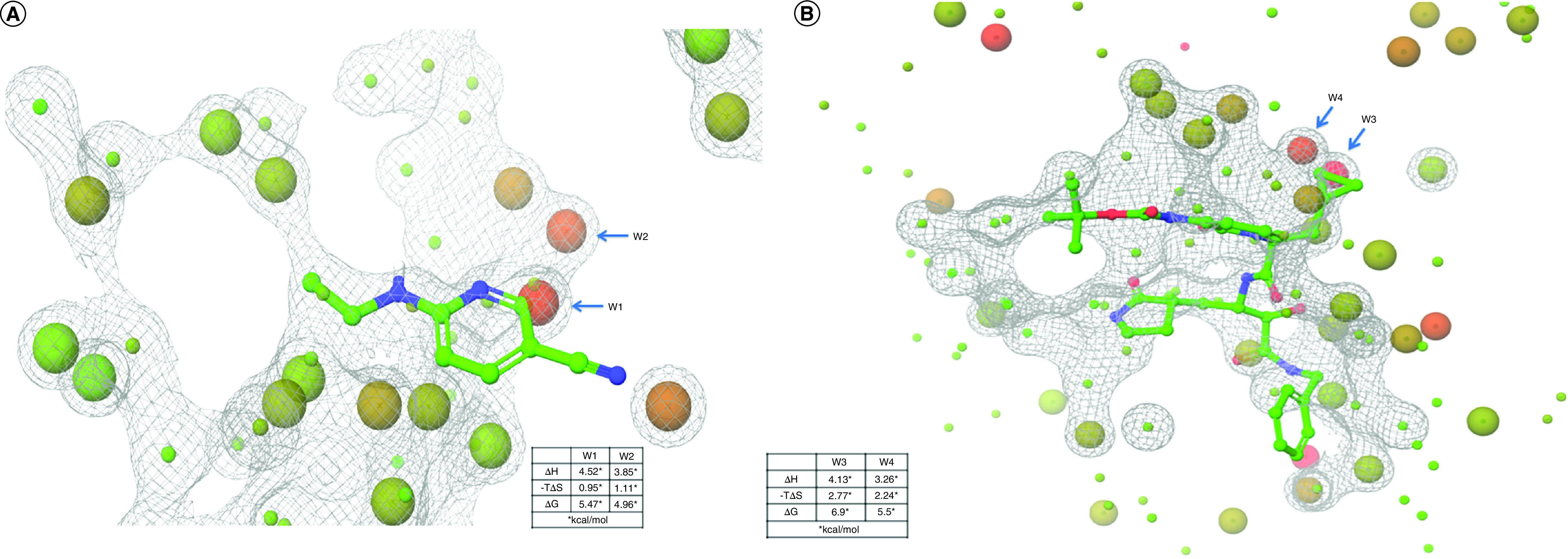
Water maps for 5 Å cavity of (A) 5R82 and (B) 6Y2G. Unstable waters are shown in red balls and reference ligands are shown as ball and stick.

The overlap of the ligand with unstable hydration sites calculated through WaterMap was examined, and it was ascertained that the selected ligand does not interfere, or minimally interferes, with the stable hydration sites. The WaterMap calculations used to determine hydration sites in the ligand binding site aided in the screening process. It calculates the enthalpic and entropic details of water clusters, which helps to score the hit molecules with diverse cores. Visual inspection of hydration sites, combined with the calculated scores, gave crucial information for the identification of potential high-affinity binders. In the present study, WaterMap calculations were executed for the prepared protein structures 5R82 and 6Y2G^32^. The generated water maps with the molecular surface for the two proteins are shown in Figure 4A & B. Only the hydration sites that fall within 5 Å of ligand binding sites were considered for analysis. The displaceable hydration sites having both ΔG and ΔH >>0 kcal/mol are identified as hydrophobic regions, which can be favorably displaced with suitable hydrophobic groups of ligands. It is noted that the displacement of such hydration sites substantially contributes to the binding of the ligands. Analysis of the water map of the 5R82 binding site showed two unstable waters in the vicinity of the pyridine ring of the ligand, which can be displaced either with methyl groups or halogens to make favorable interactions with the neighboring residues (Figure 4a). The enthalpy (ΔH), entropy (ΔS) and free energy (ΔG) for the two water molecules, W1 and W2, were found to be 4.52, 0.95 and 5.47 and 3.85, 1.11 and 4.96 kcal/mol, respectively.

Hydration sites or waters with ΔG >∼0 and ΔH <<0 kcal/mol can be considered for replacement with suitable functional groups that can make strong enthalpic interactions with the protein. These water molecules interact with protein residues through hydrophilic interactions but pay an entropic penalty for this. Thus these sites, when replaced with polar functional groups as part of the ligands, can replace the polar interactions between the protein and water molecules. There are several such hydration sites suitable for replacement with polar groups (Figure 4A & B & Supplementary Tables 1 & 2). There are also waters with ΔG <<0, which are difficult to displace or replace but can make water-bridged interactions with proteins. Also, the most stable hydration sites with ΔG <<0 (shown as bright green balls in Figure 4) are a part of the conserved regions of the protein and should be avoided while designing molecules.

In 5R82 there is a small scaffold in the binding site which can be further explored using the thermodynamic properties of the hydration sites for designing new molecules, whereas in structure 6Y2G the ligand is bigger and occupies the whole binding site cavity (Figure 3B). Also, in 6Y2G, there are two unstable water molecules observed at a distance of 2 Å from the propyl group and these sites can be further explored. The two unstable waters, W3 and W4, have enthalpy (ΔH), entropy (−TΔS) and free energy (ΔG) values as 4.13, 2.77 and 6.9 and 3.26, 2.24, 5.5 kcal/mol, respectively. There are a few more hydration sites that can be considered for replacement as per the given thermodynamic parameters. It was observed that many of the screened molecules had significant overlap with the reference ligand, and a few of them showed a more significant binding affinity in terms of the calculated parameters.

**Figure 5.**
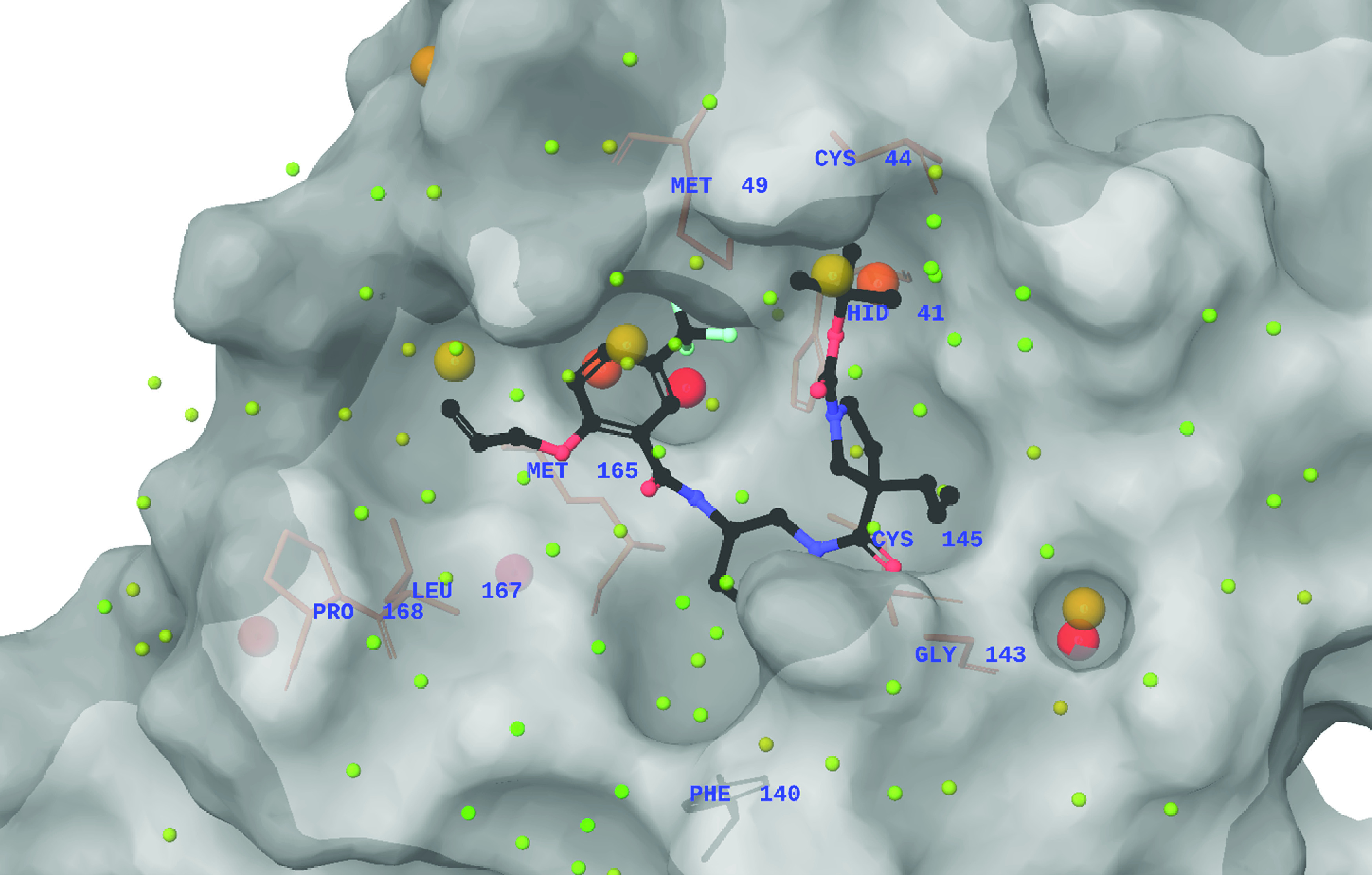
Positioning of a top-scoring Enamine molecule in the WaterMap showing the unstable waters in the binding site pockets.

An MM-GBSA scoring protocol was used to predict the binding free energies of the top 100 screened ligands, as these are expected to correlate better with the experimental results. The WaterMap score was supplemented with the terms from the MM-GBSA scoring function to derive a WM/MM score [35]. The visual analysis showed the molecules having high WM/MM ΔG scores had their hydrophobic groups hit the high-energy hydration sites. The molecules that occupied the binding site cavity, well forming potential interactions with the binding site residues (especially His41, Glu166 and Cys145) and which represented proper poses as compared with the reference ligand were selected as the final set of molecules. Figure 5 shows the WaterMap for the top-scoring molecule (Z3649466605) from the Enamine database. The WaterMap reveals the proximity of Met 49 and Met 165, sandwiching the phenyl group that hits the two unstable water molecules. Similarly, there are also two high- and medium-energy hydration sites in the His41 binding S2 pocket, hit by a propyl group of the molecule, making it a potential hit.

The obtained 362 molecules from four databases were diverse and thus gave us a good set of molecules for further systematic analysis. A set of five representative molecules was chosen from each database (Table 1) using the described strategy. The molecules were selected based on their MM-GBSA ΔG, WaterMap ΔG and WM/MM scores. Also, each molecule was analyzed for its binding pose and interactions with the functional residues of the protein. The molecules which had binding poses similar to reference ligand or covered the binding cavity efficiently were preferred over the rest. The molecules with hydrogen bonds or electrostatic interactions with functional residues like Cys145 or His41 were preferred during the selection. Other residues that were usually involved in hydrogen bond interactions were Gly143, Ser144, His163, His164 and Glu166. The interaction of the ligand with the hydration sites was also considered an important factor for the selection. For instance, the first molecule from the Enamine database, Z3649466605, hits the unstable hydration sites from the S2 and S3 pocket of the enzyme with tertiary butyl and phenyl rings, which makes it a ligand of choice. The MM-GBSA and WM/MM scores for this molecule are also comparable to those of the reference ligand, making it a suitable candidate molecule. A set of 20 promising molecules were separately analyzed for their binding properties. One of these molecules, identified from the screening of DrugBank database DB04882 (edotecarin) is a DNA topoisomerase I inhibitor of the indolocarbazole class and is being investigated for its antineoplastic activity [36]. Another identified molecule DB13764 (monoxerutin), a bioflavonoid closely related to rutin, is a promising vasoprotective agent [37]. Ligands ritonavir, ZINC67912528, ZINC67902943, DB01661, Z3464484385, Z3062886118, ZINC08455412 and ZINC08384151 showed interactions with both the residues of catalytic dyad.

**Table 1.** List of scores obtained for representative molecules from extra precision docking, molecular mechanics–generalized Born surface area and WaterMap calculations.

Sr. No.	Molecule ID	XP GScore	Water Map ΔG (kcal/mol)	Water Map enthalpy ΔH (kcal/mol)	Water Map entropy -TΔS (kcal/mol)	MM-GBSA ΔG (kcal/mol)	WM/MM ΔG bind (kcal/mol)
-	REF-LIG-6Y2G	-8.547	-29.157	1.884	-31.042	-109.77	-48.254
**Natural Products database**							
1		-10.827	-37.321	-5.654	-31.667	-100.14	-54.907
2		-9.828	-31.042	-3.071	-27.971	-95.76	-47.396
3		-11.083	-24.998	0.053	-25.051	-104.16	-46.969
4		-10.397	-31.582	-9.430	-22.152	-83.08	-44.410
5		-13.325	-25.615	-0.835	-24.782	-92.20	-42.009
**Drug Bank database**							
1		-13.719	-32.703	-1.736	-30.967	-85.86	-53.774
2		-12.167	-28.477	0.545	-29.021	-92.99	-50.464
3		-11.959	-24.571	4.376	-28.937	-83.12	-46.06
4		-11.849	-23.372	2.981	-26.353	-99.91	-41.939
5		-13.639	-25.135	6.093	-31.228	-83.64	-41.329
**Enamine database**							
1		-7.579	-37.603	-7.445	-30.157	-105.96	-53.387
2		-7.365	-30.893	-9.714	-21.179	-84.81	-46.475
3		-7.751	-24.894	-0.021	-24.873	-89.08	-41.144
4		-8.545	-30.527	-8.289	-22.238	-76.1	-40.595
5		-7.060	-20.343	3.005	-23.348	-90.80	-38.523
**Specs database**							
1		-6.897	-35.468	-7.373	-28.094	-85.81	-49.372
2		-7.829	-30.100	-6.276	-23.824	-84.45	-46.380
3		-7.611	-30.849	-3.845	-27.005	-80.16	-45.052
4		-8.29	-28.22	-4.854	-23.365	-80.57	-41.422
5		-8.466	-22.037	-0.592	-21.445	-80.24	-37.713
**Antivirals**							
1	Ritonavir	-8.246	-35.632	-0.968	-34.664	-100.95	-52.473
2	Remdesivir	-5.799	-27.258	0.936	-28.194	-76.6	-38.846
3	Hydroxychloroquine	-5.083	-20.539	-2.672	-17.867	-75.07	-32.388
4	Favipiravir	-4.732	-11.816	-5.099	-6.717	-29.05	-18.683
5	Lopinavir	-4.226	-24.963	4.932	-29.895	-86.11	-39.044

MM-GBSA: Molecular mechanics–generalized Born surface area; WM/MM: Combined WaterMap/MM-GBSA scoring protocol.

While most of the selected molecules displayed only π–π stacking interaction with His41 of the target protein, selected molecule ZINC67902943 had π–π stacking as well as hydrogen bond interaction with His41. Ligand DB01661 showed two hydrogen bonds with His41 of the protein. In addition, ligand ZINC08455412 showed a unique π–cation interaction with His41. The binding scores of the antiviral drug ritonavir were comparable to those of the reference ligand, which is in concurrence with the experimental evidence of its activity against SARS-CoV-2. The ligands that interacted with Cys145 did so through a hydrogen bond with the backbone amide. The selected molecules with the highest WM/MM binding scores fit snugly into the binding pocket. The 2D interaction diagram for these best fitting molecules is given in Supplementary Figure 1.

The strategy followed in the study yielded many potential molecules representing different chemical classes. Molecular descriptors calculation for selected molecules was also carried out using the QikProp module of the Schrödinger suite [34]. Table 1 provides a list of scores obtained from XP docking, MM-GBSA and WaterMap calculations of the selected molecules. The scores for all 362 screened molecules are given in Supplementary Table 1. The QikProp results for the representative molecules are shown in Supplementary Table 2.

## Conclusion

Herein we report a set of newly identified molecules screened by a fast combinatorial strategy using the structural information available for the novel coronavirus SARS-CoV-2. The 400 top-scoring molecules obtained from XP docking were subjected to MM-GBSA and WaterMap calculations, and stringent criteria were followed for the final selection of the molecules. Several molecules identified from the natural product database are promising and can be considered for future studies. The molecules screened from Drug Bank can be investigated for identifying repurposable drug candidates. The Enamine and Specs database molecules could be useful for researchers working in small-molecule drug discovery. The results of WaterMap calculations used in the screening study and analysis give insight into the thermodynamic properties of the hydration sites in the binding site. We believe that the identified molecules in this study will pave the way for the discovery and development of drugs for fighting the COVID-19 pandemic.

## Future perspective

The development of effective therapy to tackle the COVID-19 pandemic is the greatest challenge. Drugs originally developed to combat other viral illnesses have proven to be of little or no benefit. Rapid and robust research methods are required for the development and evaluation of new treatments. Other than the launched vaccines, there are several more in the pipeline whose effectiveness is subject to their potential to elicit antibody production. In this study, we conducted a structure-based search to identify ligands that can inhibit the M^pro^ of SARS-CoV-2. Future studies of the compounds derived through such studies are essential in order to obtain potential candidates against SARS-CoV-2. A better understanding of the role of the different viral proteins in the entry, replication or development of the virus can help scientists discover new drugs.

**Executive summary**

- Two high-resolution SARS-CoV-2 M^pro^ crystal structures and four small molecule databases were prepared for the study.
- A three-tiered docking protocol was followed to screen out ligands with high affinity toward the target protein.
- All the top molecules from the docking study were subjected to molecular mechanics–generalized Born surface area (MM-GBSA) and combined WaterMap/MM-GBSA scoring.
- WaterMap was generated for the chosen protein structures to identify different hydration sites in the ligand binding region.
- The final poses obtained after WM/MM scoring were analyzed for their binding patterns and binding pose.
- Five representative molecules that could act as potential inhibitors were reported from each database.
- Finally, the absorption, distribution, metabolism and excretion parameters were calculated for the selected molecules.
- The compounds reported in the results section may be considered for further experimental studies.
