# Supplementary material for: Rapid structure-based identification of potential SARS-CoV-2 main protease inhibitors
Source: Future Med Chem. 2021 Jun 25:10.4155/fmc-2020-0264. doi: 10.4155/fmc-2020-0264 (PMC8240652; doi:10.4155/fmc-2020-0264)
Supplement: Supplementary file 1 [file Supplementary_materials.pdf]

Supplementary Table S1. List of scores obtained for 362 molecules

Sr. No.	Molecule Id	XP GScore	Water Map ΔH Enthalpy (Kcal/mol)	Water Map ΔG (Kcal/mol)	Water Map Entropy - T ΔS (Kcal/mol)	MMGBSA ΔG (Kcal/mol)	WM/MM ΔG Bind (Kcal/mol)
	Ref-lig-6Y2G complex	-8.547	1.884	-29.157	-31.042	-109.77	-48.254
	**Natural Products Database**						
1	ZINC67910471	-10.827	-5.654	-37.321	-31.667	-100.14	-54.907
2	ZINC67910479	-9.275	-3.262	-34.826	-31.564	-99.87	-54.03
3	ZINC67912526	-10.329	-10.289	-38.052	-27.763	-73.82	-48.555
4	ZINC67912589	-9.989	-9.628	-34.984	-25.357	-80.14	-47.549
5	ZINC67903197	-10.446	-5.293	-31.034	-25.741	-90.38	-47.463
6	ZINC67910753	-9.828	-3.071	-31.042	-27.971	-95.76	-47.396
7	ZINC67912528	-11.083	0.053	-24.998	-25.051	-104.16	-46.969
8	ZINC15112222	-7.333	-5.488	-30.27	-24.782	-87.09	-45.919
9	ZINC67902754	-9.664	-3.614	-37.345	-33.731	-68.2	-45.803
10	ZINC15676330	-7.626	-1.831	-25.929	-24.097	-83.17	-44.927
11	ZINC38143828	-10.296	2.209	-27.824	-30.034	-93.02	-44.789
12	ZINC31167288	-10.397	-9.43	-31.582	-22.152	-83.08	-44.41
13	ZINC31167292	-10.397	-9.43	-31.582	-22.152	-83.08	-44.41
14	ZINC08624572	-8.355	0.392	-23.415	-23.807	-91.09	-42.718
15	ZINC20465660	-7.139	1.913	-23.951	-25.864	-97.2	-42.717
16	ZINC49898130	-10.886	-5.593	-29.591	-23.998	-83.56	-42.567
17	ZINC01650233	-8.615	-5.212	-30.161	-24.949	-77.8	-42.303
18	ZINC67903429	-9.166	3.402	-29.598	-32.999	-84.66	-42.067
19	ZINC67902943	-13.325	-0.835	-25.617	-24.782	-92.2	-42.009
20	ZINC67903024	-10.752	-7.348	-30.086	-22.738	-69.36	-41.916
21	ZINC38143830	-10.436	3.366	-25.63	-28.997	-90.84	-41.673
22	ZINC67911281	-9.278	4.279	-24.844	-29.123	-91.27	-41.384
23	ZINC04216676	-9.146	3.447	-24.714	-28.161	-89.06	-41.344
24	ZINC67902879	-11.363	5.81	-21.974	-27.784	-96.5	-41.212
25	ZINC15676365	-7.125	-0.407	-22.109	-21.702	-87.15	-41.185
26	ZINC59677186	-7.085	-6.513	-32.649	-26.136	-65.62	-40.961
27	ZINC08643408	-8.43	-9.624	-28.043	-18.419	-83.18	-40.706
28	ZINC67911491	-8.191	6.564	-25.795	-32.36	-85.35	-40.49
29	ZINC67913779	-9.92	-8.583	-34.167	-25.584	-62.13	-40.283
30	ZINC67910479	-11.017	4.331	-27.779	-32.11	-84.02	-40.171
31	ZINC67902469	-12.815	1.828	-21.733	-23.561	-98.68	-40.131
32	ZINC67912581	-9.116	-2.468	-29.527	-27.058	-73.42	-39.823
33	ZINC38139530	-10.881	3.003	-25.858	-28.861	-82.11	-39.223
34	ZINC04096846	-12.151	5.16	-25.003	-30.163	-79.81	-39.105
35	ZINC33861403	-10.47	-0.856	-24.63	-23.774	-74.05	-38.748
36	ZINC35455223	-9.454	0.382	-23.404	-23.786	-82.21	-38.671
37	ZINC67911292	-11.669	-2.713	-24.426	-21.713	-85.29	-38.532
38	ZINC31167122	-10.166	-2.459	-24.313	-21.854	-73.82	-38.229
39	ZINC67911231	-11.147	5.255	-21.894	-27.149	-81.41	-37.967
40	ZINC67903231	-9.133	-4.479	-27.662	-23.182	-92.05	-37.862
41	ZINC05433680	-7.086	-3.529	-26.132	-22.603	-66.64	-37.531
42	ZINC67910695	-8.915	7.391	-24.465	-31.857	-78.71	-37.347
43	ZINC38139797	-8.65	7.385	-22.023	-29.407	-85.66	-37.268
44	ZINC31153552	-7.558	-11.62	-30.324	-18.704	-54.09	-37.202
45	ZINC67903429	-7.131	4.056	-24.666	-28.722	-76.52	-36.722

46	ZINC67902657	-12.791	0.107	-29.144	-29.251	-65.82	-36.471
47	ZINC15675744	-7.308	6.299	-18.912	-25.211	-81.67	-35.17
48	ZINC67912583	-8.188	5.034	-25.209	-30.243	-60.18	-35.063
49	ZINC04098556	-10.341	5.388	-19.734	-25.122	-77.01	-34.853
50	ZINC35456617	-7.265	-7.303	-26.107	-18.804	-63.01	-34.833
51	ZINC67911489	-10.483	10.408	-18.821	-29.229	-85.44	-34.751
52	ZINC00844289	-8.159	-3.075	-21.928	-18.853	-66.44	-34.74
53	ZINC67902860	-9.072	4.409	-23.407	-27.816	-71.44	-34.734
54	ZINC67912395	-9.326	1.53	-27.608	-29.138	-55.65	-34.364
55	ZINC15676330	-7.277	3.131	-17.956	-21.087	-74.01	-33.821
56	ZINC38139526	-11.136	3.896	-26.648	-30.544	-62.38	-33.809
57	ZINC67912393	-10.267	1.558	-24.814	-26.372	-69.54	-33.671
58	ZINC49898792	-11.538	9.776	-16.364	-26.14	-82.31	-33.438
59	ZINC67903250	-10.113	7.729	-20.62	-28.35	-70.31	-33.402
60	ZINC12360166	-7.083	5.459	-15.968	-21.428	-80.31	-32.883
61	ZINC35455270	-9.615	1.007	-20.485	-21.492	-78.37	-32.325
62	ZINC38143762	-11.142	6.956	-23.16	-30.116	-73.5	-32.323
63	ZINC05433814	-7.015	3.945	-18.252	-22.198	-75.95	-32.12
64	ZINC67902883	-10.571	8.419	-16.501	-24.921	-80.88	-32.09
65	ZINC30879455	-7.909	1.128	-21.834	-22.962	-68.04	-31.85
66	ZINC31165292	-11.152	3.261	-19.067	-22.328	-71.97	-31.835
67	ZINC67912659	-8.423	3.847	-19.956	-23.804	-75.97	-31.726
68	ZINC67903538	-8.907	-2.616	-31.542	-28.926	-34.95	-30.905
69	ZINC02106570	-6.971	-6.417	-18.673	-12.256	-50.46	-30.532
70	ZINC14687876	-7.699	0.112	-17.852	-17.964	-69.5	-29.492
71	ZINC01639355	-7.881	-4.343	-19.011	-14.668	-56.14	-27.781
72	ZINC14687903	-7.423	0.406	-16.641	-17.047	-64.95	-26.452
73	ZINC08627147	-8.999	7.67	-11.404	-19.074	-63.66	-21.891
74	ZINC20463331	-8.002	5.289	-14.794	-20.083	-57.93	-21.099
75	ZINC19800831	-8.019	9.084	-11.288	-20.372	-52.5	-20.396
76	ZINC36521966	-7.519	10.625	-4.523	-15.148	-57.01	-19.189
**Drug Bank Database**							
77	DB01661	-13.719	-1.736	-32.703	-30.967	-85.86	-53.774
78	DB13764	-13.639	6.093	-25.135	-31.228	-83.64	-41.329
79	DB04023	-13.111	-6.71	-30.101	-23.39	-66.09	-44.007
80	DB03041	-12.69	5.879	-18.23	-24.109	-69.4	-34.776
81	DB04158	-12.569	4.721	-24.281	-29.002	-71.59	-42.002
82	DB01792	-12.167	0.545	-28.477	-29.021	-92.99	-50.464
83	DB02547	-12.125	-2.371	-26.237	-23.866	-75.32	-43.062
84	DB01698	-12.064	2.146	-25.834	-27.98	-35.1	-24.537
85	DB12410	-12.035	-10.556	-32.697	-22.14	-80.09	-44.571
86	DB02358	-12.026	-4.526	-26.422	-21.896	-66.75	-41.665
87	DB03186	-11.959	4.366	-24.571	-28.937	-83.12	-46.06
88	DB04882	-11.849	2.981	-23.372	-26.353	-99.91	-41.939
89	DB04099	-11.826	8.984	-16.431	-25.415	-75.97	-32.974
90	DB03397	-11.796	7.906	-13.972	-21.877	-84.6	-32.536
91	DB04226	-11.718	4.28	-16.659	-20.939	-71.45	-28.631
92	DB01690	-11.689	-0.554	-22.686	-22.131	-35.49	-29.227
93	DB02059	-11.299	0.164	-22.789	-22.953	-67.98	-37.181
94	DB03461	-11.134	13.817	-12.647	-26.464	-62.5	-24.127
95	DB03755	-11.079	11.188	-9.565	-20.753	-70.11	-28.195
96	DB03956	-11.079	12.41	-3.957	-16.367	-65.24	-20.568
97	DB03296	-11.065	-7.006	-29.907	-22.9	-77.96	-43.855
98	DB00157	-10.926	13.123	-13.523	-26.645	-50.04	-23.625
99	DB02196	-10.847	-0.802	-23.273	-22.471	-84.62	-43.042
100	DB04482	-10.758	4.86	-17.97	-22.83	-70.91	-32.191
101	DB04651	-10.715	-9.302	-32.542	-23.241	-85.5	-48.953
102	DB03751	-10.626	3.481	-19.031	-22.512	-79.15	-36.428

103	DB02498	-10.617	-0.291	-30.405	-30.114	-81.55	-46.227
104	DB03161	-10.574	2.555	-18.774	-21.328	-76.93	-36.675
105	DB02554	-10.49	11.666	-12.671	-24.337	-57.07	-24.452
106	DB04418	-10.407	-3.642	-23.536	-19.895	-57.87	-37.701
107	DB02319	-10.33	11.01	-19.273	-30.283	-63.56	-30.22
108	DB08237	-10.303	-0.498	-25.207	-24.709	-72.03	-40.122
109	DB04983	-10.219	9.652	-17.795	-27.447	-51.67	-29.678
110	DB03797	-10.21	13.714	-14.346	-28.06	-65.7	-27.927
111	DB04421	-10.207	2.078	-18.932	-21.01	-47	-26.645
112	DB01975	-10.148	10.943	-12.142	-23.085	-64.13	-26.522
113	DB03687	-10.137	-1.841	-23.529	-21.689	-68.08	-38.223
114	DB03022	-10.128	-12.082	-31.555	-19.473	-83.23	-51.623
115	DB03532	-10.041	-2.013	-21.218	-19.205	-54.73	-33.858
116	DB02338	-9.882	11.082	-14.635	-25.717	-63.36	-28.628
117	DB11616	-9.882	-12.525	-33.347	-20.822	-66	-40.74
118	DB11670	-9.855	3.774	-20.336	-24.109	-74.25	-33.763
119	DB12052	-9.851	1.836	-23.396	-25.232	-84.18	-37.759
120	DB01960	-9.802	-1.685	-18.407	-16.722	-60.89	-32.682
121	DB02629	-9.774	-0.272	-34.744	-34.472	-91.3	-48.515
122	DB04097	-9.692	2.498	-17.795	-20.293	-65.47	-31.126
123	DB02557	-9.658	2.415	-24.407	-26.822	-73.77	-37.433
124	DB12937	-9.602	-2.549	-20.68	-18.131	-47.17	-28.683
125	DB04408	-9.591	5.581	-26.136	-31.717	-89.65	-40.947
126	DB00158	-9.5	4.192	-20.607	-24.799	-68.92	-34.261
127	DB04514	-9.499	-3.727	-26.015	-22.288	-65.54	-39.98
128	DB02732	-9.462	8.235	-19.802	-28.037	-60.55	-30.326
129	DB02651	-9.439	3.381	-26.094	-29.475	-69.35	-38.656
130	DB15246	-9.434	-0.345	-26.583	-26.239	-57.87	-35.516
131	DB03300	-9.427	7.532	-13.923	-21.455	-74.32	-30.756
132	DB01177	-9.39	-2.033	-25.057	-23.024	-76.83	-38.78
133	DB03812	-9.379	7.072	-15.142	-22.213	-58.42	-27.039
134	DB01864	-9.344	2.493	-20.615	-23.108	-66.64	-38.116
135	DB04121	-9.263	13.951	-5.048	-18.999	-65.83	-22.747
136	DB08185	-9.26	0.758	-21.832	-22.59	-83.55	-37.36
137	DB03962	-9.234	7.163	-19.151	-26.314	-80.12	-38.386
138	DB11184	-9.119	-1.557	-25.919	-24.362	-77.05	-39.098
139	DB04396	-9.042	-6.321	-23.951	-17.63	-78.66	-38.196
140	DB03714	-9.017	-2.758	-24.378	-21.619	-78.31	-42.249
141	DB02098	-8.955	2.9	-15.97	-18.87	-63.79	-33.145
142	DB12665	-8.949	7.068	-15.519	-22.587	-73.22	-30.906
143	DB09421	-8.893	-2.827	-20.307	-17.48	-60.25	-30.772
144	DB03638	-8.827	-0.271	-23.78	-23.509	-55.47	-32.17
145	DB04461	-8.808	-2.095	-27.865	-25.77	-78.13	-41.122
146	DB01831	-8.746	5.149	-14.265	-19.414	-66.76	-30.307
147	DB05616	-8.746	-4.296	-24.52	-20.223	-61.84	-35.665
148	DB04096	-8.719	-2.342	-20.921	-18.579	-77.32	-38.141
149	DB01893	-8.711	3.857	-21.547	-25.405	-71.18	-37.202
150	DB13490	-8.679	-0.446	-29.197	-28.751	-62.54	-37.194
151	DB11296	-8.672	2.288	-19.411	-21.699	-53.39	-29.348
152	DB07409	-8.548	-2.801	-20.938	-18.137	-70.72	-35.656
153	DB03160	-8.503	-4.456	-26.774	-22.318	-87.8	-44.392
154	DB04438	-8.481	2.199	-13.307	-15.505	-45.41	-20.522
155	DB00452	-8.458	8.667	-17.711	-26.378	-58.28	-25.526
156	DB12757	-8.371	-3.911	-28.751	-24.84	-71.51	-40.881
157	DB03823	-8.363	4.1	-12.036	-16.136	-49.16	-20.783
158	DB03247	-8.347	7.357	-12.943	-20.3	-56.01	-22.008
159	DB02224	-8.34	-6.25	-20.933	-14.683	-55.63	-31.735
160	DB14772	-8.307	-6.311	-25.25	-18.939	-72.61	-38.279

161	DB04789	-8.305	-4.438	-25.956	-21.518	-64.41	-38.461
162	DB14636	-8.299	0.662	-16.651	-17.313	-67.37	-32.52
163	DB02483	-8.266	4.139	-26.037	-30.176	-76.95	-40.855
164	DB03973	-8.242	5.372	-17.919	-23.292	-58.87	-30.473
165	DB02082	-8.238	9.887	-11.719	-21.606	-54.71	-25.94
166	DB01700	-8.181	7.036	-9.154	-16.19	-43.83	-20.114
167	DB12441	-8.16	9.308	-18.852	-28.16	-63.64	-28.936
168	DB02540	-8.153	8.623	-16.863	-25.487	-81.1	-35.365
169	DB06866	-8.117	-11.335	-31.89	-20.555	-72.49	-41.697
170	DB03148	-8.094	7.164	-8.934	-16.098	-55.96	-22.526
171	DB12895	-8.048	5.254	-25.983	-31.237	-87.07	-38.705
172	DB12977	-8.004	-2.522	-28.719	-26.197	-92.09	-44.729
173	DB00224	-7.998	-6.325	-30.784	-24.459	-86.5	-42.537
174	DB01995	-7.985	2.008	-14.087	-16.096	-60.08	-28.845
175	DB01812	-7.955	-0.034	-17.477	-17.442	-55.61	-32.194
176	DB02333	-7.953	4.386	-16.301	-20.687	-70.71	-36.131
177	DB04014	-7.945	-4.369	-19.83	-15.461	-53.86	-29.253
**Enamine Database**							
178	Z1899461190	-8.545	-8.289	-30.527	-22.238	-76.1	-40.595
179	Z1344189646	-8.327	-6.603	-23.029	-16.426	-62.05	-31.338
180	Z1951768720	-8.141	-2.212	-18.583	-16.371	-63.04	-31.387
181	Z1443065604	-8.123	1.452	-18.715	-20.167	-71.58	-31.455
182	Z2194546487	-7.88	1.809	-18.151	-19.96	-54.94	-28.023
183	Z1748052226	-7.797	-0.265	-15.304	-15.04	-48.09	-21.314
184	Z1443065604	-7.702	-1.487	-24.023	-22.536	-73.57	-36.393
185	Z1899473976	-7.693	-7.092	-25.618	-18.525	-69.41	-34.139
186	Z3635404747	-7.677	-1.688	-21.617	-19.929	-81.57	-37.083
187	Z1698911037	-7.666	-3.201	-24.049	-20.849	-72.81	-34.834
188	Z1522274835	-7.654	-4.202	-22.368	-18.166	-72.61	-31.65
189	Z3649466605	-7.579	-7.445	-37.603	-30.157	-105.96	-53.387
190	Z3062886118	-7.562	-0.021	-24.894	-24.873	-89.08	-41.144
191	Z3242334951	-7.552	-6.245	-21.41	-15.164	-48.36	-30.563
192	Z1217457777	-7.54	-6.121	-22.649	-16.529	-75.67	-37.101
193	Z1904515500	-7.489	-2.213	-20.561	-18.348	-70.61	-34.207
194	Z2798426411	-7.489	-6.046	-25.12	-19.074	-75.5	-34.868
195	Z3040093352	-7.486	-13.404	-29.144	-15.741	-67.08	-41.673
196	Z1422257462	-7.476	-2.633	-21.559	-18.926	-72.9	-33.017
197	Z1688669918	-7.431	-4.512	-24.099	-19.588	-65.89	-37.626
198	Z2788449789	-7.414	-7.166	-26.74	-19.573	-73.34	-39.032
199	Z3210945312	-7.373	-6.137	-19.138	-13.001	-63.4	-28.463
200	Z3464484385	-7.365	-9.714	-30.893	-21.179	-84.81	-46.475
201	Z1932170341	-7.363	0.936	-20.157	-21.093	-80.98	-34.125
202	Z2873129857	-7.361	-5.168	-17.721	-12.553	-58.14	-27.176
203	Z2482017188	-7.358	-0.737	-18.772	-18.035	-46.59	-27.115
204	Z1411786551	-7.337	-4.395	-26.777	-22.382	-71.45	-38.545
205	Z3664687470	-7.33	-5.089	-23.324	-18.235	-62.54	-34.683
206	Z1522025926	-7.275	-4.598	-21.013	-16.415	-69.88	-29.436
207	Z1870021901	-7.259	-9.535	-23.244	-13.709	-48.81	-31.332
208	Z770161780	-7.254	-0.582	-19.826	-19.244	-58.62	-29.423
209	Z1370795512	-7.247	-9.423	-26.616	-17.193	-52.09	-31.263
210	Z1723437505	-7.229	-1.477	-22.125	-20.648	-75.65	-37.358
211	Z1442891551	-7.228	-4.482	-22.77	-18.288	-72.28	-35.229
212	Z1615665435	-7.213	-1.751	-20.194	-18.443	-72.81	-32.54
213	Z1141894386	-7.211	-8.659	-23.951	-15.292	-53.33	-32.825
214	Z3615437914	-7.196	-9.196	-30.019	-20.823	-79.07	-45.632
215	Z1747853639	-7.192	-3.563	-23.351	-19.788	-65.64	-36.053
216	Z1870021901	-7.15	-5.863	-21.612	-15.749	-44.9	-28.079
217	Z1984431740	-7.149	-5.8	-23.265	-17.465	-45.27	-30.438

218	Z2961486187	-7.144	-2.464	-24.435	-21.971	-78.99	-38.653
219	Z2528665912	-7.092	-6.614	-23.01	-16.396	-47.71	-29.689
220	Z1315856390	-7.087	-2.902	-19.678	-16.775	-57.77	-30.355
221	Z1229943565	-7.08	-7.76	-27.824	-20.064	-70.03	-41.075
222	Z3015132220	-7.075	-2.906	-23.821	-20.915	-59.32	-31.814
223	Z2788447560	-7.058	-6.591	-22.601	-16.01	-61.05	-33.263
224	Z1474147457	-7.052	-4.213	-22.65	-18.437	-68.1	-35.445
225	Z2959870623	-7.042	-2.5	-20.986	-18.486	-63.84	-33.03
226	Z1723430650	-7.012	-3.605	-26.395	-22.79	-65.6	-37.719
227	Z1474144239	-7.003	-9.524	-25.019	-15.494	-62.58	-38.626
228	Z2044060381	-6.997	-4.987	-24.866	-19.88	-63.31	-30.818
229	Z2061541512	-6.982	-2.752	-20.323	-17.57	-64.82	-31.398
230	Z1331536923	-6.972	-5.227	-20.216	-14.989	-59.52	-31.609
231	Z2788449789	-6.97	-5.933	-26.9	-20.967	-67.32	-37.59
232	Z1869984537	-6.958	-4.322	-23.859	-19.538	-67.48	-34.173
233	Z2005156965	-6.956	0.373	-18.337	-18.71	-68.55	-31.223
234	Z3041580464	-6.941	-5.837	-24.045	-18.209	-62.11	-32.642
235	Z341776266	-6.928	-5.638	-26.261	-20.623	-60.08	-38.306
236	Z1444778233	-6.889	-4.061	-21.492	-17.431	-36.07	-23.496
237	Z2178138706	-6.889	-11.408	-26.936	-15.529	-51.67	-37.112
238	Z3062879805	-6.877	3.414	-17.947	-21.361	-80.46	-32.013
239	Z1497727961	-6.875	-4.396	-23.87	-19.474	-64.62	-34.762
240	Z751406438	-6.874	-0.328	-22.913	-22.585	-90.95	-37.46
241	Z1748054572	-6.873	-6.717	-21.545	-14.828	-58.57	-32.631
242	Z3062887599	-6.871	3.005	-20.343	-23.348	-90.8	-38.523
243	Z2087848899	-6.836	-1.803	-16.816	-15.013	-51.63	-25.535
244	Z341776266	-6.835	-2.856	-24.086	-21.229	-58.24	-35.685
245	Z1972782733	-6.829	-0.731	-14.406	-13.674	-66.01	-27.008
246	Z1025446834	-6.82	-3.804	-15.714	-11.909	-57.46	-27.115
247	Z1974971668	-6.799	-1.747	-25.827	-24.08	-73.87	-39.872
248	Z849671072	-6.776	-13.31	-32.025	-18.715	-47.19	-36.349
249	Z1890693129	-6.743	-1.146	-16.358	-15.212	-59.38	-26.589
250	Z1890693129	-6.743	-1.146	-16.358	-15.212	-59.38	-26.589
251	Z2972322701	-6.725	4.304	-14.29	-18.594	-58.92	-24.283
252	Z1265558714	-6.715	-1.88	-23.966	-22.087	-79.73	-37.548
253	Z3290580018	-6.715	-0.827	-18.208	-17.381	-69.91	-31.445
254	Z1609531046	-6.711	-4.057	-24.977	-20.92	-65.78	-35.474
255	Z3062884818	-6.693	3.444	-18.227	-21.67	-85.63	-35.327
**Specs Database**							
256	ZINC00641197	-8.466	-0.592	-22.037	-21.445	-80.24	-37.713
257	ZINC00706449	-8.391	-4.394	-25.996	-21.602	-71.59	-39.018
258	ZINC00679320	-8.315	-5.344	-27.361	-22.018	-69.93	-40.764
259	ZINC00641249	-8.307	1.713	-15.894	-17.607	-75.04	-29.738
260	ZINC08384151	-8.29	-4.854	-28.22	-23.365	-80.57	-41.422
261	ZINC36044798	-8.224	0.455	-21.027	-21.483	-76.03	-34.404
262	ZINC13942028	-8.132	-13.557	-36.324	-22.767	-25.57	-34.831
263	ZINC00641233	-8.112	-0.443	-20.703	-20.26	-77.9	-36.424
264	ZINC06645813	-8.089	-4.629	-25.896	-21.267	-88.78	-39.522
265	ZINC00706470	-8.083	-0.245	-23.475	-23.23	-79.71	-35.236
266	ZINC00971085	-8.051	-1.886	-22.669	-20.783	-70.92	-36.493
267	ZINC00641227	-8.015	-0.816	-21.155	-20.338	-79.37	-37.025
268	ZINC00847278	-7.991	-2.315	-21.329	-19.014	-67.63	-34.763
269	ZINC00641225	-7.984	-1.633	-22.565	-20.932	-76.44	-36.97
270	ZINC00641211	-7.966	1.363	-17.479	-18.842	-71.71	-31.821
271	ZINC13551169	-7.962	-2.637	-23.17	-20.533	-84.32	-36.969
272	ZINC00182977	-7.957	-2.464	-21.193	-18.729	-65.26	-33.972
273	ZINC00725460	-7.929	-5.029	-26.002	-20.972	-70.45	-39.076
274	ZINC00641153	-7.889	1.434	-17.256	-18.69	-71.3	-31.758

275	ZINC00862555	-7.848	-4.239	-23.95	-19.711	-69.4	-36.482
276	ZINC01016443	-7.848	0.778	-18.361	-19.139	-74.84	-33
277	ZINC00725466	-7.841	-3.464	-23.098	-19.634	-65.92	-35.494
278	ZINC04371961	-7.829	-6.276	-30.1	-23.824	-84.45	-46.38
279	ZINC00641209	-7.805	1.239	-17.624	-18.863	-73.25	-31.867
280	ZINC00641219	-7.786	0.609	-18.585	-19.194	-75.18	-33.027
281	ZINC00641164	-7.775	1.475	-17.301	-18.776	-75.67	-32.635
282	ZINC00862810	-7.725	5.093	-14.441	-19.534	-68.49	-28.108
283	ZINC19597204	-7.721	-9.745	-30.605	-20.861	-55.28	-37.82
284	ZINC00706519	-7.693	2.176	-13.537	-15.713	-63.58	-25.248
285	ZINC00634911	-7.679	1.702	-15.173	-16.875	-56.88	-26.32
286	ZINC00985688	-7.677	-2.413	-23.672	-21.258	-80.98	-38.569
287	ZINC05785575	-7.669	-1.239	-21.506	-20.267	-78.16	-35.94
288	ZINC00706470	-7.661	0.941	-20.341	-21.282	-66.78	-33.905
289	ZINC02187844	-7.653	0.188	-19.865	-20.054	-79.95	-32.793
290	ZINC02085622	-7.643	-4.531	-26.075	-21.544	-77.7	-38.753
291	ZINC00725553	-7.619	0.463	-16.03	-16.493	-62.61	-27.353
292	ZINC08455412	-7.611	-3.845	-30.849	-27.005	-80.12	-45.052
293	ZINC02085787	-7.585	0.013	-24.433	-24.447	-76.95	-35.547
294	ZINC02747018	-7.58	-2.214	-22.848	-20.633	-83.22	-37.78
295	ZINC00845573	-7.527	4.634	-16.501	-21.135	-71.6	-30.729
296	ZINC04066384	-7.51	3.326	-12.968	-16.294	-69.76	-25.017
297	ZINC02104352	-7.504	-3.691	-23.855	-20.164	-81.71	-37.605
298	ZINC02742156	-7.501	2.921	-19.387	-22.307	-69.57	-29.062
299	ZINC13555009	-7.489	-2.514	-29.008	-26.493	-70.44	-39.698
300	ZINC00993349	-7.446	-4.489	-27.775	-23.286	-80.1	-40.871
301	ZINC13144609	-7.434	10.543	-7.151	-17.694	-23.04	-7.882
302	ZINC00071321	-7.431	0.02	-16.405	-16.425	-62.24	-25.358
303	ZINC00845585	-7.415	5.451	-13.702	-19.153	-66.07	-27.084
304	ZINC01009870	-7.406	0.135	-20.285	-20.42	-85.29	-34.828
305	ZINC00708949	-7.397	4.548	-15.224	-19.772	-70.87	-29.265
306	ZINC13424220	-7.393	-7.53	-25.601	-18.071	-65.92	-36.106
307	ZINC00706477	-7.384	-3.067	-21.371	-18.304	-74.45	-33.276
308	ZINC04066632	-7.377	1.149	-16.794	-17.943	-71.47	-27.83
309	ZINC02771690	-7.368	-2.248	-22.266	-20.018	-69.83	-34.01
310	ZINC04030866	-7.334	-0.385	-17.63	-17.245	-75.18	-30.278
311	ZINC01926532	-7.287	-4.308	-20.934	-16.626	-48.68	-28.573
312	ZINC08834685	-7.284	4.53	-16.456	-20.986	-95.53	-35.048
313	ZINC12726073	-7.281	-5.102	-24.671	-19.569	-66.16	-37.231
314	ZINC02920577	-7.258	-1.338	-19.215	-17.877	-70.41	-30.783
315	ZINC08384321	-7.255	-3.28	-29.217	-25.937	-78.61	-42.257
316	ZINC02695916	-7.248	-0.854	-21.314	-20.46	-70.61	-33.328
317	ZINC04476085	-7.242	8.06	-7.685	-15.745	-60.22	-19.168
318	ZINC04619065	-7.23	2.954	-20.032	-22.986	-61.76	-28.275
319	ZINC00845657	-7.22	5.526	-13.192	-18.718	-62.52	-25.974
320	ZINC00974500	-7.216	-3.823	-23.164	-19.341	-72.99	-36.251
321	ZINC00298364	-7.211	-0.535	-19.083	-18.548	-61.85	-30.236
322	ZINC04364878	-7.204	1.027	-21.783	-22.81	-67.21	-35.684
323	ZINC02187934	-7.193	1.719	-18.843	-20.562	-72.98	-29.923
324	ZINC00684207	-7.188	-2.912	-22.41	-19.499	-78.22	-35.855
325	ZINC02186823	-7.165	-4.066	-23.293	-19.227	-75.68	-33.984
326	ZINC02095801	-7.127	2.721	-17.356	-20.078	-72.4	-29.403
327	ZINC06757348	-7.099	-1.701	-20.912	-19.21	-70.33	-29.908
328	ZINC19801929	-7.093	-10.733	-32.635	-21.902	-59.24	-40.273
329	ZINC04369149	-7.068	0.871	-21.846	-22.717	-77.63	-38.136
330	ZINC00716301	-7.066	-2.165	-21.078	-18.912	-65.08	-32.945
331	ZINC04064575	-7.065	-5.996	-27.134	-21.138	-66.78	-38.739
332	ZINC05769485	-7.041	-8.154	-24.408	-16.254	-45.48	-31.906

333	ZINC02745365	-7.033	0.056	-21.904	-21.96	-77.33	-33.604
334	ZINC02914960	-6.993	0.15	-18.693	-18.843	-74.95	-31.031
335	ZINC04631885	-6.983	-2.777	-19.337	-16.56	-60.47	-31.462
336	ZINC04066269	-6.973	-1.863	-20.016	-18.153	-68.92	-29.928
337	ZINC01212264	-6.971	4.318	-17.21	-21.528	-85.15	-34.16
338	ZINC13123003	-6.957	-4.653	-18.973	-14.32	-58.15	-29.674
339	ZINC00639154	-6.939	-6.19	-32.202	-26.012	-76.11	-44.208
340	ZINC00712983	-6.938	-9.34	-28.535	-19.195	-68.57	-44.627
341	ZINC17111482	-6.933	0.57	-13.287	-13.857	-56.44	-24.164
342	ZINC05825739	-6.928	-1.158	-19.649	-18.491	-73.61	-34.034
343	ZINC00664731	-6.926	0.761	-22.556	-23.318	-66.82	-35.78
344	ZINC02478992	-6.911	-6.018	-18.458	-12.44	-47.43	-24.681
345	ZINC08455009	-6.897	-7.373	-35.468	-28.094	-85.81	-49.372
346	ZINC06386085	-6.896	-2.06	-20.986	-18.926	-74.97	-35.774
347	ZINC00644398	-6.891	0.565	-29.025	-29.59	-86.79	-43.332
348	ZINC00816767	-6.88	3.522	-14.6	-18.122	-67.68	-25.775
349	ZINC02912026	-6.872	-0.111	-18.243	-18.132	-71.53	-29.686
350	ZINC04019931	-6.869	-4.076	-22.884	-18.808	-71.35	-34.41
351	ZINC06570965	-6.853	-2.885	-19.353	-16.467	-68.71	-28.6
352	ZINC08449631	-6.833	-11.537	-34.36	-22.823	-64.57	-38.657
353	ZINC04473112	-6.819	1.81	-20.931	-22.741	-57.1	-28.751
354	ZINC09088346	-6.817	-11.193	-29.485	-18.291	-79.98	-42.672
355	ZINC00632469	-6.802	-4.893	-25.764	-20.871	-82.75	-41.195
356	ZINC00654256	-6.791	1.445	-24.146	-25.591	-73.01	-36.664
**Antivirals**							
357	Ritonavir	-8.246	-0.968	-35.632	-34.664	-100.95	-52.473
358	Remdesvir	-5.799	0.936	-27.258	-28.194	-76.6	-38.846
359	Azithromycin	-5.621	8.156	-9.888	-18.044	-78.91	-24.028
360	Hydroxychlorquinine	-5.083	-2.672	-20.539	-17.867	-75.07	-32.388
361	Favipiravir	-4.732	-5.099	-11.816	-6.717	-29.05	-18.683
362	Lopinovir	-4.226	4.932	-24.963	-29.895	-86.11	-39.044

Supplementary Table S2. QikProp results of ADME parameters of top-ranked molecules screened from different databases

Sr.No	Molecule Id	S	M.W	QPlogPo/w	QPlogS	QPPCaco	QPlogBB	PHOA	ROF
**Natural Product Database**									
1	ZINC67910471	7	662.643	1.698	-5.487	7.781	-4.395	13.958	3
2	ZINC67910753	11	686.663	-2.055	-2.006	3.036	-4.662	0	3
3	ZINC67912528	7	622.668	1.992	-3.305	7.112	-3.964	14.979	3
4	ZINC31167288	3	514.531	4.198	-6.946	36.406	-2.971	66.512	1
5	ZINC67902943	7	564.499	-1.889	-2.818	3.155	-4.153	0	3
**Drug Bank Database**									
1	DB01661	10	719.279	-3.203	0.159	0	-6.977	0	3
2	DB01792	9	653.392	-3.643	-1.821	0.001	-6.404	0	3
3	DB03186	12	797.352	-2.877	-1.087	0	-8.001	0	3
4	DB04882	7	608.56	-1.602	-3.207	0.848	-4.669	0	3
5	DB13764	10	654.577	-2.35	-2.912	0.44	-5.801	0	3
**Enamine Database**									
1	Z3649466605	1	565.632	6.13	-8.089	1757.979	-0.423	94.999	2
2	Z3464484385	0	375.872	4.032	-5.04	1103.025	-0.448	100	0
3	Z3062886118	0	398.507	2.896	-4.657	733.834	-0.866	95.189	0
4	Z1899461190	0	446.468	4.027	-5.633	1723.086	0.053	100	0
5	Z3062887599	0	383.492	3.326	-4.969	1442.076	0.411	100	0
**Specs Database**									
1	ZINC18455009	0	575.396	4.453	-6.958	608.725	-0.694	89.899	1
2	ZINC04371961	0	404.471	3.111	-5.387	323.924	-1.424	90.094	0
3	ZINC08455412	2	450.856	0.953	-4.699	7.766	-3.404	35.5	1
4	ZINC08384151	0	440.9	3.711	-5.891	457.052	-1.013	96.281	0
5	ZINC00641197	0	441.483	4.899	-6.284	905.915	-0.957	100	0
**Antivirals**									
1	Ritonavir	9	720.943	6.828	-8.364	385.644	-1.859	74.338	3
2	Remdesvir	4	602.583	1.344	-4.737	37.111	-3.117	36.991	2
3	Hydroxychlorquinine	0	335.876	3.173	-2.903	442.502	-0.207	92.882	0
4	Favipiravir	1	157.104	-0.402	-1.247	109.87	-0.968	61.119	0
5	Lopinovir	3	628.81	5.691	-6.255	781.339	-1.404	86.124	2

QikProp parameters for ADME: S (STARS) = Number of property/descriptor values falling outside the 95% range of similar values for known drugs (Recommended value 0 - 5) ; MW = Molecular weight; QPlogPo/w = Predicted octanol/water partition coefficient (Recommended values –2.0 - 6.5); QPlogS = Predicted aqueous solubility (Recommended values –6.5 - 0.5); QPPCaco = Predicted apparent Caco-2 cell permeability in nm/sec (Recommended values <25 poor, >500 great); QPlogBB = Predicted brain/blood partition coefficient (Recommended values –3.0 - 1.2); PHOA= Predicted human oral absorption on 0 to 100% ROF = Number of violations to rule of five

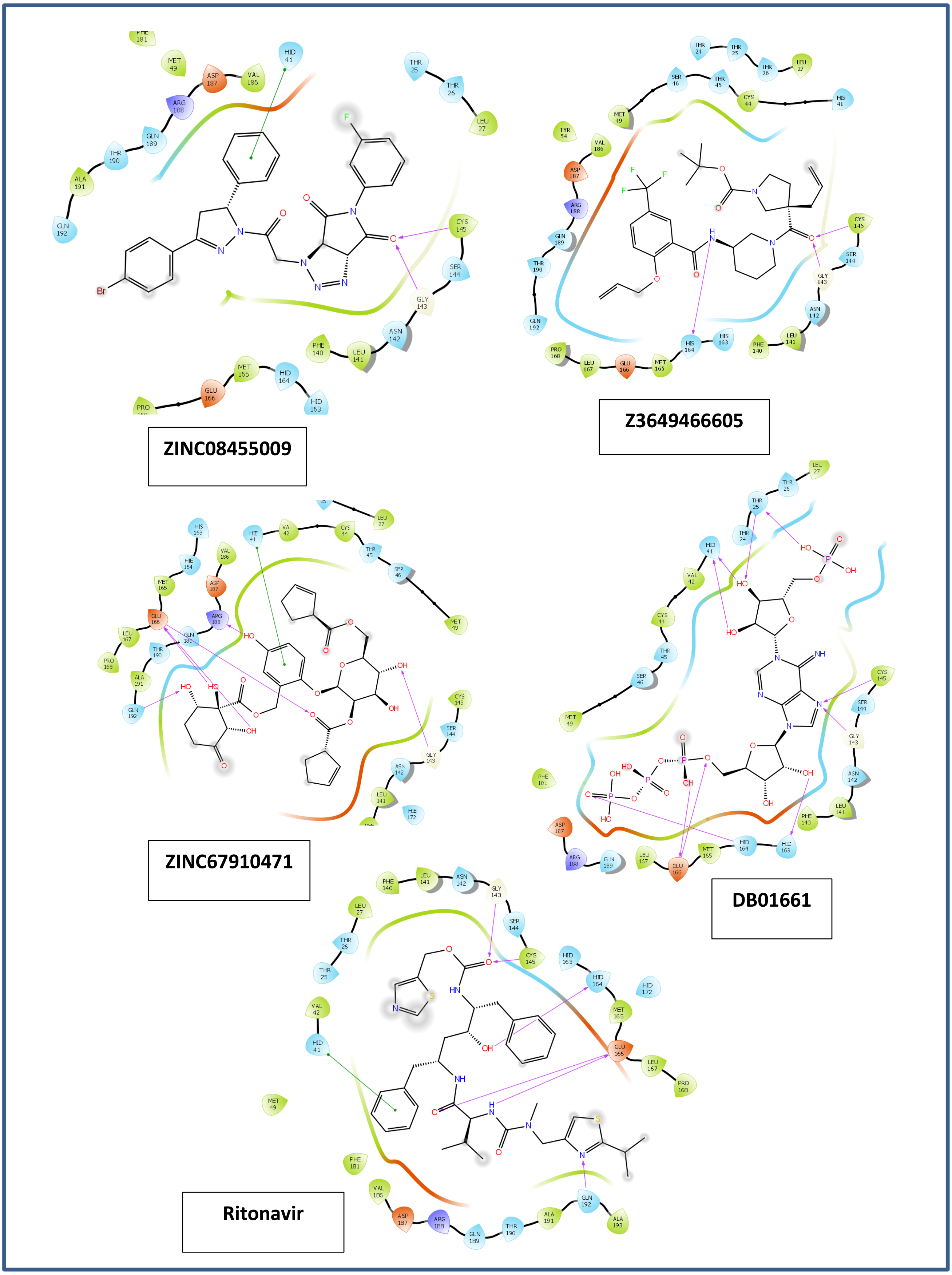

Supplementary Figure S1. 2D interaction diagrams of selected molecules
